# Rus-GXF, a ruscogenin glycoside, binds to the ADP-binding domain of JAK1 to prevent inflammation and barrier damage in acute lung injury

**DOI:** 10.3389/fphar.2026.1730503

**Published:** 2026-03-10

**Authors:** Long Liu, Xu Duan, Mei-Qi Li, Yi Gu, Jia-He Zhao, Kai-Shuai Si, Nan-Nan Wang, Xing-Fu Chen, Zhong-Qiong Yin, Li-Xia Li, Xun Zhou, Chun Wu, Meng-Liang Tian, Yuan-Feng Zou

**Affiliations:** 1 Natural Medicine Research Center, College of Veterinary Medicine, Sichuan Agricultural University, Chengdu, China; 2 Key Laboratory of Animal Disease and Human Health of Sichuan Province, College of Veterinary Medicine, Sichuan Agricultural University, Chengdu, China; 3 College of Agronomy, Sichuan Agricultural University, Chengdu, China; 4 College of Science, Sichuan Agricultural University, Ya’an, China

**Keywords:** acute lung injury, inflammation, JAK1/STAT3, kinase inhibitor, molecule

## Abstract

**Background:**

Ruscogenin-1-O-[β-D-glucopyranosyl (1→2)][β-D-xylopyranosyl (1→3)] -β-D-fucopyranoside (Rus-GXF) is a ruscogenin glycoside of *Liriope muscari* (Decaisne) L. H. Bailey, yet its protective effects against acute lung injury—a condition characterized by exacerbated inflammation and barrier damage have not been fully elucidated.

**Methods:**

In this study, the preventive and therapeutic effects of Rus-GXF on acute lung injury (ALI) were investigated using transcriptome RNA sequencing, network pharmacology, molecular docking, molecular dynamics simulation and other *in vitro* and *in vivo* experiments.

**Results:**

Rus-GXF suppressed inflammatory responses in two key cell types involved in lung injury. In immune cells (RAW264.7), it inhibited the production of pivotal pro-inflammatory mediators and their regulatory genes. Similarly, in pulmonary epithelial cells (BEAS-2B), it reduced the expression of inflammatory signals and concurrently enhanced markers of cellular tight junction proteins. In mice, Rus-GXF alleviated ALI severity, evidenced by decreased lung wet/dry ratio, bronchoalveolar lavage fluid protein content, pro-inflammatory cytokine levels, and histopathological scores. Integrated network pharmacology and transcriptomics indicated that Rus-GXF acts through multi-target mechanisms in ALI. Molecular docking and dynamics simulations revealed that Rus-GXF acts as an allosteric inhibitor of JAK1, thereby preventing its activation and subsequent STAT3 phosphorylation. The inhibitory effect of Rus-GXF on the JAK1/STAT3 signaling pathway was investigated by immunohistochemistry (IHC) and Western blot analysis (WB).

**Conclusion:**

These results demonstrate that Rus-GXF suppresses the macrophage-derived cytokine storm, alleviates inflammation, and improves barrier function. It functions as a JAK1 inhibitor to regulate ALI progression via the JAK1/STAT3 signaling pathway.

## 1 Introduction

Acute lung injury (ALI) is characterized by increased inflammatory exudation in the alveolar space, resulting in lung tissue edema and impaired gas exchange. Cytokine storm, an abnormal immune response to infections, mediates the development of ALI and leads to severe adverse effects. The main cytokines of cytokine storm include interleukin (IL), tumor necrosis factor (TNF) and colony stimulating factor (CSF). These overproduced cytokines aggravate pathological damage to organs through positive feedback (Zhang et al., 2023). Therefore, reducing the excessive production of inflammatory factors during ALI can be effective means of preventing and treating ALI. The JAK/STAT signaling pathway is particularly important in the regulation of cytokine storms (Li et al., 2024). Various related cytokines bind to membrane receptors, resulting in JAK becoming an activated form, which can be well docked with STATs and mediate the phosphorylation of STATs (Luo et al., 2020). Upon phosphorylation, STATs form dimers that enter the nucleus and are transported to the target gene promoter region, thereby regulating the transcription and production of inflammatory cytokines (Clark et al., 2014).

However, ALI exhibits significant therapeutic limitations. Although glucocorticoids have potent anti-inflammatory effects, prolonged administration may induce severe adverse effects, including immunosuppression, secondary infections, and hyperglycemia (Zimmerman et al., 2021). Non-steroidal anti-inflammatory drugs (NSAIDs) exhibit limited efficacy in mitigating cytokine storms and may potentially exacerbate renal impairment (Black, 2019). Moreover, excessive antibiotic administration disrupts pulmonary microbiome equilibrium and potentiates antimicrobial resistance development (Ventola, 2015). Although traditional Chinese medicine (TCM) formulations exhibit multi-target therapeutic potential, they face challenges, including compositional complexity and quality control difficulties (Hua et al., 2021). These limitations underscore the urgent need to develop novel ALI treatment strategies.

In contrast, monomeric metabolites derived from TCM offer distinct advantages. First, their well-defined chemical structures enable precise quality control and standardized production, effectively addressing the batch-to-batch variability associated with complex TCM formulations (Rampazzo et al., 2018). Second, these metabolites exert multi-target effects by simultaneously modulating key pathological processes in ALI, including inflammatory, oxidative stress, and apoptosis. For instance, baicalin has been shown to attenuate alveolar damage by inhibiting NLRP3 inflammasome activation (Wang et al., 2023). Third, their well-characterized pharmacokinetic profiles facilitate dose optimization and the management of toxicity. Notably, tanshinone IIA demonstrates significant pulmonary protective effects through the activation of the Nrf2/HO-1 pathway and has a favorable safety profile (Chen et al., 2020).

According to traditional Chinese medicine theory, *L. muscari* (Decaisne) L. H. Bailey possesses protective effects on the lungs. Owing to this property, it is commonly used as an metabolite in medicinal diets and suggests its potential value in treating specific modern diseases. Moreover, its saponin metabolites, characterized by a sweet taste profile, have garnered increasing attention in the food industry. Ruscogenin-1-O-[β-D-glucopyranosyl (1→2)][β-D-xylopyranosyl (1→3)] -β-D-fucopyranoside (Rus-GXF), the predominant steroidal saponin metabolites isolated from *L. muscari*, is a key quality control marker for this medicinal botanical drug. Rus-GXF exhibits strong anti-inflammatory, immunopharmacological and cardioprotective activities (Li et al., 2014). However, its potential therapeutic effects on ALI require further investigation.

Despite the promising therapeutic potential of natural products such as Rus-GXF in inflammatory diseases, a significant knowledge gap remains concerning its specific role and mechanistic action in ALI. To address this, our study proposes an integrated strategy that synergistically combines network pharmacology, transcriptomics, and molecular docking. This approach advances beyond conventional single-target hypotheses by employing network pharmacology to predict the multi-target therapeutic profile of Rus-GXF. Furthermore, transcriptomics provides unbiased experimental validation, confirming its modulatory effects on key signaling pathways in an ALI model. Finally, molecular docking offers atomic-level mechanistic insights by visualizing and assessing the plausible binding modes between Rus-GXF and its core predicted targets.

## 2 Materials and methods

### 2.1 Materials and reagents

Rus-GXF was obtained from Aliddin Co., Ltd. (L418618, CAS: 87480-46-4, Shanghai, China), and its purity is more than 98%. Lipopolysaccharide (LPS, *Escherichia coli* 055: B5) was obtained from Sigma-Aldrich (Merck KGaA, Darmstadt, Germany). The RAW264.7 and BEAS-2B cells were obtained from the Chinese Academy of Sciences Institution (Chinese Academy of Sciences, Guangzhou, China). Commercial kits (IL-6, IL-1β, TNF-α, NO) were supplied by the Nanjing Jiancheng Bioengineering Institute (Nanjing, China). The primer of Tumor Necrosis Factor-α (TNF-α), Interleukin-6 (IL-6), Cyclooxygenase-2 (COX2), Zonula Occludens-1 (ZO-1), Occludin, C-X-C Motif Chemokine Ligand 1 (CXCL1), C-X-C Motif Chemokine Ligand 2 (CXCL2), and β-actin were purchased from Invitrogen Co., Ltd. (Shanghai, China). All antibodies related to the experiment (iNOS, TNF-α, p-STAT3, STAT3, JAK1, GAPDH) were purchased from Cell Signaling Technology (Shanghai, China). Fetal bovine serum and other reagents for cell treatment were purchased from Gibco/BRL Life (Grand Island, NY, United States).

### 2.2 Cell culture

Macrophage RAW264.7 and Bronchial Epithelium transformed with Ad12-SV40 2B (BEAS-2B) were placed in a cell incubator with 5% CO_2_ at 37 °C, and cultured in high glucose DMEM medium containing 10% fetal bovine serum and 1% double antibody (penicillin, streptomycin). The medium was refreshed regularly, and cells were passaged upon reaching confluence. The cells in logarithmic growth phase were taken for experiments.

### 2.3 Screening of Rus-GXF concentration

Cell viability was assessed by CCK-8 assay. RAW264.7 and BEAS-2B cells were seeded in 96-well plates (1 × 10^5^ cells/mL), excluding the outermost wells filled with PBS. After 6 h, cells were treated with serially diluted Rus-GXF (RAW264.7: 0, 1, 10, 20, 40, 60 μM; BEAS-2B: 0, 1, 10, 20, 40, 80, 160 μM; *n* = 3) for 24 h. Then, 110 μL of CCK-8 working solution (CCK-8/complete medium = 1/10; Beijing Solarbio Science & Technology Co., Ltd.) was added per well. Following 2 h of incubation in the dark, absorbance at 450 nm was measured using a microplate reader (Thermo Fisher Scientific, Inc.).

### 2.4 Determination of NO, IL-6 and TNF-α in RAW264.7 supernatant

Based on the results of the CCK-8 assay, non-cytotoxic concentrations of Rus-GXF were selected for the subsequent drug administration experiments. RAW264.7 cells were seeded in 48-well plates at a density of 1 × 10^5^/mL. After 6 h of adherence, cells were treated according to the following groups for 24 h: blank control group (Control), model group (1 μg mL^-1^ LPS) and administration group (40 μM Rus-GXF + 1 μg mL^-1^ LPS) (Xu et al., 2022). After 24 h of cell culture, the cell culture medium was centrifuged at 2,500 rpm × min^−1^ and 4 °C for 10 min. The supernatant was taken according to the operation of the corresponding kit to determine the secretion of NO, IL-6 and TNF-α.

### 2.5 Determination of gene expression by quantitative real-time PCR

RAW264.7 (40 μM Rus-GXF) and BEAS-2B (80 μM Rus-GXF cells were cultured, followed by mRNA extraction, reverse transcription with 5×Evo M-MLV RT Master Mix, and qPCR using TransStart Top Green qPCR SuperMix on a CFX96 Touch™ system (BIO-RAD, CA, United States). Relative quantification analysis was performed using the 2^−ΔΔCq^ method with GAPDH used as the reference gene (Livak and Schmittgen, 2001). The primer sequences are listed in Table 1.

**TABLE 1:** Primer sequence used in quantitative real-time PCR.

Cell	Gene	Primer sequence (5′to 3′)
RAW264.7	COX2	F: TTC​AAC​ACA​CTC​TAT​CAC​TGG​C
		R: AGA​AGC​GTT​TGC​GGT​ACT​CAT
	iNOS	F: TCA​CGC​TTG​GGT​CTT​GTT​CA
		R: CCT​TTT​CCT​CTT​TCA​GGT​CAC​TT
	β-actin	F: GTG​GCA​TCC​ATG​AAA​CTA​CAT
		R: GGC​ATA​GAG​GTC​TTT​ACG​G
BEAS-2B	IL-6	ACT​CAC​CTC​TTC​AGA​ACG​AAT​TG
		CCA​TCT​TTG​GAA​GGT​TCA​GGT​TG
	TNF-α	TCT​TCT​CCT​TCC​TGA​TCG​TG
		GCC​AGA​GGG​CTG​ATT​AGA​GA
	CXCL1	TGC​TGA​ACA​GTG​ACA​AAT​CCA​AC
		TGG​GGT​TGA​CAT​TTC​AAA​AAG​AA
	CXCL2	CTC​AAG​AAT​GGG​CAG​AAA​GC
		CTT​CAG​GAA​CAG​CCA​CCA​AT
	ZO-1	CAA​CAT​ACA​GTG​ACG​CTT​CAC​A
		CAC​TAT​TGA​CGT​TTC​CCC​ACT​C
	Occludin	AGA​CCT​GAT​GAA​TTC​AAA​CCC​A
		CCA​CAC​AGG​CAA​ATA​TGG​CG
	β-actin	AGA​GCT​ACG​AGC​TGC​CTG​AC
		AGC​ACT​GTG​TTG​GCG​TAC​AG

F, forward; R, reverse.

### 2.6 Animal experimental design and administration

Twenty-four 4-week-old healthy male ICR mice weighing about 15 g were caged in SPF animal rooms and fed with irradiated sterilized experimental mice food. All mice were permitted free access to food and water. The ambient temperature of the environment was maintained at 25 °C ± 2 °C, the humidity level was kept at 50% ± 10%, and the photoperiod was set to 12 h of light and 12 h of darkness. After 1 week of adaptation to the environment, the mice were randomly divided into 3 groups, with 8 mice in each group. The mice were divided into blank control group (Control), ALI model control group (ALI) and Rus-GXF treatment group (ALI + Rus-GXF). Mice in the Rus-GXF treatment group were administered Rus-GXF (4 mg/kg) (Zhang et al., 2017; Fan et al., 2018) via intraperitoneal injection for three consecutive days. The Rus-GXF was dissolved in a vehicle solution consisting of 5% DMSO, 30% PEG-400, and 65% saline. Mice in the Control and ALI groups received an equivalent volume of the vehicle solution on the same schedule. We established the ALI mouse model with reference to the method described by Ehrentraut et al. (2019). One hour after the final treatment, mice were anesthetized with Zoletil (Virbac, Carros, France) and challenged intratracheally with 5 mg/kg LPS dissolved in sterile phosphate-buffered saline (PBS) to induce ALI. PBS was used as the vehicle control. Once awake, the mice were transferred to the SPF observation room for routine feeding. After 8 h after modelling, animals were anesthetized with isoflurane and sacrificed by cervical dislocation in order to obtain experimental samples.

All animal experiments were approved by the Ethics Committee of Sichuan Agricultural University (Confirmation No. DYXY141641006). All animal experiments were performed in accordance with the National Institutes of Health Guide for the Care and Use of Laboratory Animals.

### 2.7 Determination of indicators in bronchoalveolar lavage fluid (BALF)

The total protein level in BALF serves as a direct marker of alveolar-capillary barrier impairment in ALI, and its elevation is used for the quantitative assessment of lung injury severity. After cervical dislocation, bronchoalveolar lavage fluid (BALF) was collected from the trachea using 0.01 mol/L PBS (0.5 mL × 3). The BALF was centrifuged (4 °C, 10 min, 3,500 r/min), and the total protein in the supernatant was quantified by a BCA kit (BL521A, Biosharp). Meanwhile, serum levels of IL-6, IL-1β, and TNF-α were determined by ELISA.

### 2.8 Determination of lung wet/dry weight ratio in mice

The lung wet/dry weight ratio is a critical indicator for quantifying the severity of pulmonary edema, a hallmark pathological feature of ALI, as it directly measures water content accumulation resulting from increased vascular permeability. The left lungs were rinsed with sterile saline, blotted dry, and weighed to obtain the wet weight. They were then dried in a 60 °C oven to constant weight to obtain the dry weight. The wet/dry (W/D) ratio was calculated to assess pulmonary edema.

### 2.9 Hematoxylin and eosin staining (HE)

HE staining serves as the gold-standard histological method for ALI assessment, enabling direct visualization and scoring of key pathological features. The lung tissues of mice in each group were collected, fixed with formalin solution, dehydrated, embedded in paraffin, made into 4 μm sections, stained with HE, and sealed with neutral gum. The pathological changes of lung tissues of mice in each group were observed under optical microscope.

### 2.10 Western blotting

Lung tissues from mice in each group were homogenized in the presence of a protease denaturation inhibitor (G2007-1ML, Servicebio, Wuhan, China), followed by total protein extraction. Protein concentration was determined using a BCA kit (BL521A, Biosharp, Hefei, China) and adjusted to uniform levels. The following primary antibodies were used: rabbit monoclonal anti-JAK1 (1:1,000), anti-STAT3 (1:2000), anti-phospho-STAT3 (1:2,000), and mouse polyclonal anti-GAPDH (1:1,000). After incubation, membranes were washed five times and then probed with horseradish peroxidase-conjugated goat anti-rabbit (1:5,000) or anti-mouse (1:5,000) IgG antibodies (Zhongshan Golden Bridge, Beijing, China) at room temperature for 1 h. Protein bands were visualized using an enhanced chemiluminescence system on a chemiluminescence imager (ChemiScope 6100, Qinxiang, Shanghai, China), and quantified by densitometry with ImageJ.

### 2.11 Immunohistochemistry

Paraffin-embedded sections (4 μm) underwent antigen retrieval in citrate buffer (pH 6.0) after deparaffinization and rehydration. Endogenous peroxidase was blocked with 3% H_2_O_2_ (15 min), followed by blocking with 5% BSA (1 h). Sections were incubated overnight at 4 °C with anti-iNOS (1:200), anti-TNF-α (1:150), or anti-p-STAT3 (Tyr705, 1:100), then with HRP-conjugated secondary antibodies (1:500, 1 h, RT). DAB staining and hematoxylin counterstaining were performed. Images were acquired using a pathological scanner and quantified with ImageJ.

### 2.12 Network pharmacology

To systematically investigate the potential mechanisms of Rus-GXF in treating pneumonia and acute lung injury (ALI), the chemical structure of Rus-GXF was first retrieved from the PubChem database (https://pubchem.ncbi.nlm.nih.gov) and subsequently submitted to the SEA (https://sea.bkslab.org), Super-PRED (https://prediction.charite.de/index.php), and GeneCards (https://www.genecards.org) databases for target prediction. Disease-related targets for pneumonia and ALI were then retrieved by searching the GeneCards (https://www.genecards.org), OMIM (https://omim.org), and TTD (https://db.idrblab.net/ttd) databases using the keywords “Pneumonia” and “Acute Lung Injury.” The common targets between Rus-GXF and the diseases were identified by constructing a Venn diagram using an online bioinformatics platform (https://www.bioinformatics.com.cn). A “Rus-GXF–pneumonia–ALI” interaction network was visualized using Cytoscape 3.10.2. To further explore protein-protein interactions (PPI), the common targets were imported into the STRING database (https://cn.string-db.org) with the species set as “*Homo sapiens*” and a minimum interaction score >0.9. The resulting PPI network was imported into Cytoscape 3.10.2 for visualization and core target screening.

Finally, functional enrichment analysis was performed using the DAVID database (https://davidbioinformatics.nih.gov). Gene Ontology (GO) and Kyoto Encyclopedia of Genes and Genomes (KEGG) pathway analyses were conducted with a significance threshold of *P* ≤ 0.05. The top 10 enriched terms in each GO category—biological process (BP), cellular component (CC), and molecular function (MF)—were selected based on ascending *P*-values and visualized using a three-category bar chart. KEGG pathway results were sorted by *P*-value and presented in an advanced bubble chart using the online platform (https://www.bioinformatics.com.cn) to systematically elucidate the potential biological functions and signaling pathways involved.

### 2.13 Transcriptome sequencing

Lung tissues from the Control, ALI, and ALI + Rus-GXF groups (*n* = 4) were randomly collected, and total RNA was extracted using Trizol Reagent (Invitrogen Life Technologies), with concentration, purity, and integrity assessed by a NanoDrop spectrophotometer (Thermo Scientific). Transcriptome libraries were constructed from 1 µg total RNA with the TruSeq™ RNA Sample Preparation Kit, followed by cDNA size selection (400–500 bp) using AMPure XP beads, PCR amplification, and purification. The resulting libraries were quantified and sequenced on an Illumina NovaSeq 6000 platform (Shanghai Personal Biotechnology Cp. Ltd.). Differential gene expression analysis between groups was performed using DESeq2, with DEGs defined as |log_2_FoldChange| > 1 and *P*-value <0.05. GO and KEGG pathway enrichment analyses were conducted on selected DEGs, and results were visualized via the Microbiotics website and Cytoscape 3.10.2.

### 2.14 Molecular docking and visualization

Molecular docking was conducted with JAK1, JAK2, and JAK3, based on their status as the key receptor-associated kinases and core components of the JAK/STAT3 signaling cascade. JAK1 (PDB ID: 6RSH), JAK2 (PDB ID: 8BXH), JAK3 (PDB ID: 7Q6H) were obtained from the PDB database. The structure of Rus-GXF were obtained from the TCMSP database (https://www.tcmsp-e.com/) in mol2 format. Docking simulations were performed using AutoDock vina1.2.5 software. The binding affinity and activity were assessed based on the docking score, and the docking outcomes were optimized and visualized using Pymol3.1 software.

### 2.15 Molecular dynamics simulation (MD)

All-atom MD simulations (200 ns, CHARMM36, 300 K) in GROMACS 2025.01 revealed conformational dynamics of JAK1 and its complex with Rus-GXF after standard system preparation and energy minimization (Shahwan et al., 2023; Naqvi et al., 2018). The analytical metrics included root-mean-square deviation (RMSD) of the ligand and backbone atoms, radius of gyration (Rg), solvent accessible surface area (SASA), root-mean-square fluctuation (RMSF) of various amino acid residues, and hydrogen bond (H-bond) count.

### 2.16 Statistical analysis

The data were presented as the mean ± standard deviation (SD) from at least three independent biological replicates, and all the statistical analyses were performed using SPSS 22.0 software. All the histograms experiments were plotted by GraphPad Prism 5.0 software. Statistical significance of the data from the control and experimental groups was compared by one-way analysis of variance (ANOVA) and the Least Significant Difference test. *P* < 0.05 was considered statistically significant. The normality of data distribution was assessed using the Shapiro-Wilk test (or Kolmogorov-Smirnov test). Only data that passed the normality test were subjected to one-way ANOVA followed by Tukey’s post hoc test.

## 3 Results

### 3.1 Rus-GXF has good anti-inflammatory activity in RAW264.7

**FIGURE 1:**
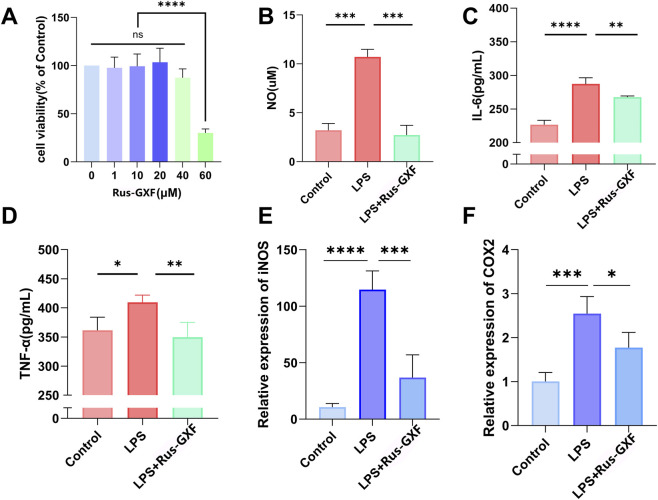
Anti-inflammatory effect of monomeric Rus-GXF on LPS-induced RAW264.7 cells. **(A)** CCK-8 assay of Rus-GXF on RAW264.7 cells (*n* = 6). **(B–D)** Cell culture supernatant NO, IL-6 and TNF-α content assay (*n* = 4). **(E,F)** Real-time fluorescence quantitative PCR assay to detect the relative gene expression of COX2 and iNOS (*n* = 4). Bars are expressed as mean ± SD. *P < 0.05; **P < 0.01; ***P < 0.001.

Since CCK-8 experiments demonstrated that RAW 264.7 growth was not significantly impacted by Rus-GXF in the dose range of 0–40 μM, we chose to use the administration concentration of 40 μM for the subsequent experiments. However, a higher concentration of Rus-GXF (60 μM) significantly inhibited the growth of RAW 264.7 cells, leading to a reduction in cell viability of more than 50% (Figure 1A). Rus-GXF was shown to effectively attenuate the LPS-induced increase in the secretion levels of cellular NO, IL-6, and TNF-α by 20%–60% (Figures 1B–D). Furthermore, it reduced the mRNA levels of COX-2 and iNOS in RAW 264.7 cells, with a reduction ranging from 30% to 60% (Figures 1E,F).

### 3.2 Rus-GXF ameliorates barrier function and attenuates inflammatory response in BEAS-2B

**FIGURE 2:**
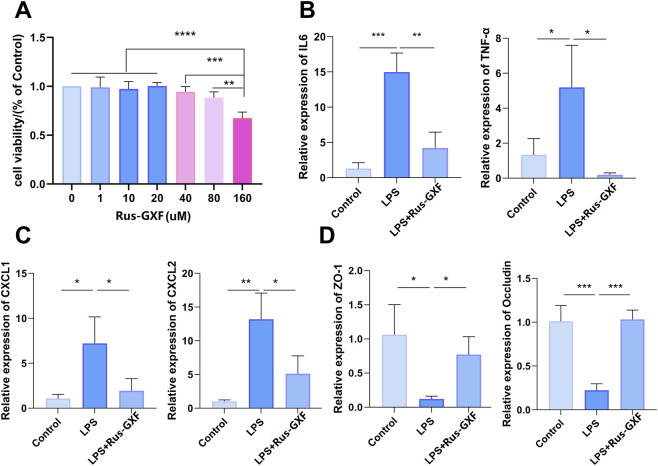
Modulation of inflammatory responses and barrier function by Rus-GXF in BEAS-2B cells. **(A)** CCK-8 assay of Rus-GXF on BEAS-2B cells (*n* = 6). **(B)** Real-time fluorescence quantitative PCR assay to detect the relative gene expression of IL-6 and TNF-α (*n* = 4). **(C)** The gene expression of CXCL1 and CXCL2. **(D)** The gene expression of ZO-1 and Occludin (*n* = 4). Bars are expressed as mean ± SD. *P < 0.05; **P < 0.01; ***P < 0.001.

Our findings indicated that Rus-GXF exhibited significant cytotoxicity against BEAS-2B cells at a concentration of 160 μΜ, reducing cell viability by 30% (Figure 2A). Therefore, a concentration of 80 μM was selected for all subsequent cellular experiments. LPS treatment induced a series of inflammatory responses and compromised barrier function in airway epithelial cells. We found that Rus-GXF treatment suppressed the gene expression of both inflammatory cytokines (Figure 2B) and the chemokines CXCL1 and CXCL2 (Figure 2C), with a uniform suppression rate of 40%–70%. Furthermore, LPS impaired the barrier function of BEAS-2B cells, as evidenced by reduced expression of the tight junction proteins ZO-1 and Occludin (Figure 2D), and this impairment was effectively restored by a more than fourfold increase following Rus-GXF treatment.

### 3.3 Rus-GXF can protect ALI mice

**FIGURE 3:**
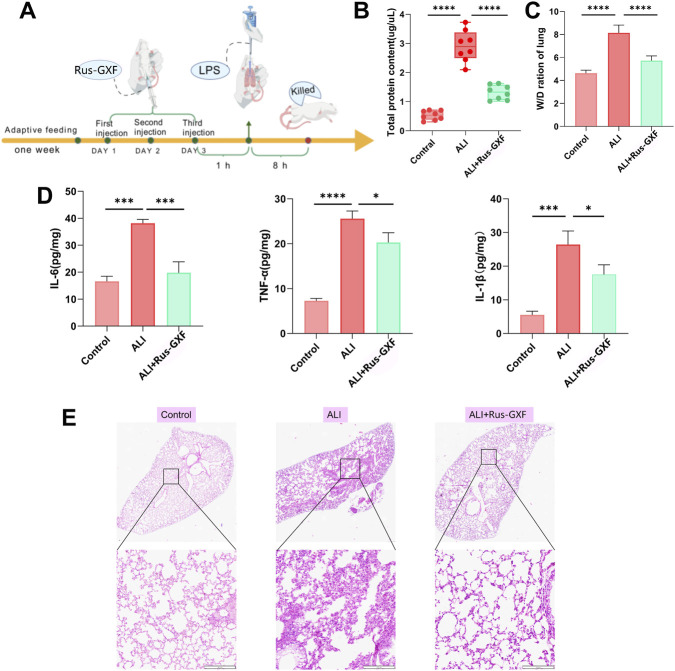
Rus-GXF’s effect on ALI mice. **(A)** Techniques for modeling ALI. **(B)** Alveolar lavage fluid’s total protein content (*n* = 6). **(C)** Lung tissue’s wet-dry weight (*n* = 6). **(D)** Alveolar lavage fluid IL-6, TNF-α, and IL-1β concentrations (*n* = 6). **(E)** H&E staining indicates injury to lung tissue (*n* = 3). Bars are expressed as mean ± SD. *P < 0.05; **P < 0.01; ***P < 0.001.

The flow chart displays ALI mice modeling (Figure 3A). Early administration of Rus-GXF was found to effectively reduce the levels of inflammatory cytokines in alveolar lavage fluid (Figure 3D), as evidenced by the decrease in the total protein concentration in alveolar lavage fluid and the wet-to-dry weight ratio of lung tissues (Figures 3B,C). Rus-GXF could effectively reduce pathological alterations such inflammatory cellular infiltration brought on by LPS and disarray of the lungs’ alveolar structure (Figure 3E).

### 3.4 Rus-GXF intervenes in the ALI process through multiple targets and pathways

**FIGURE 4:**
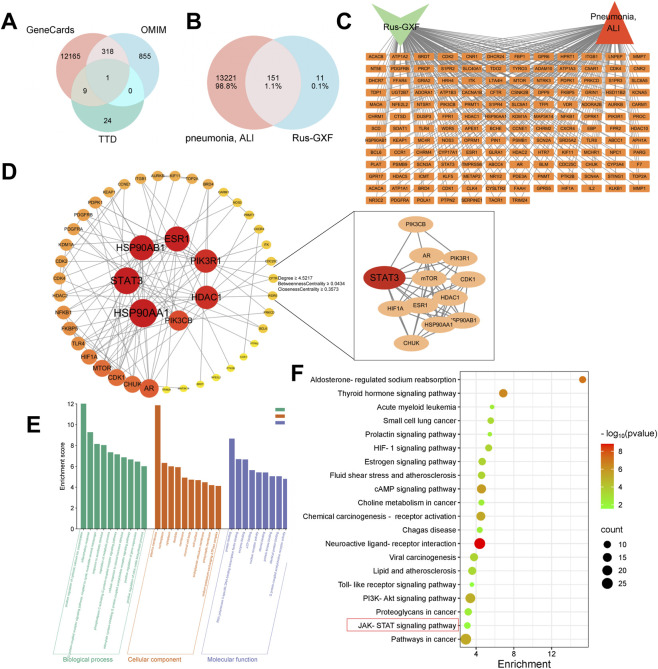
Network pharmacology approaches to find Rus-GXF targets, pathways, and functions. **(A)** ALI target collection of three databases. **(B)** Disease-Rus-GXF Wayne diagram. **(C)** Metabolite-target-ALI linkage diagram. **(D)** Core target screening. **(E)** GO enrichment analysis. **(F)** KEGG enrichment analysis.

Target prediction for Rus-GXF was conducted by screening public databases, yielding a total of 162 potential targets. Following data integration and removal of duplicates, 13,372 unique disease-related targets were identified (Figure 4A). The targets of pneumonia, ALI and Rus-GXF were integrated and 151 intersection targets were obtained (Figure 4B). The obtained 151 intersection targets were imported into Cytoscape3.10.2 software for integration, and the ' Rus-GXF-Pneumonia, ALI ' network relationship was established (Figure 4C). There are 153 nodes and 301 edges in the figure. Subsequently, the 151 common targets were imported into the STRING database. A proteins network was then constructed with Cytoscape 3.10.2, and 93 interconnected core targets were identified from this network. The circle was the target. The larger the graph, the deeper the color, and the greater the degree value. Twelve key genes, such as STAT3, ESR1, HIF1A, and others, were found by screening (Figure 4D). According to GO enrichment analysis, Rus-GXF can influence nuclear and cytoplasmic cell membranes, the inflammatory response, positive regulation of gene expression, enzyme binding, and protein binding, all of which can mitigate ALI (Figure 4E). KEGG enrichment analysis showed that the JAK/STAT pathway was significantly enriched (Figure 4F).

### 3.5 Transcriptomic profiling confirms in volvement of the JAK/STAT pathway

**FIGURE 5:**
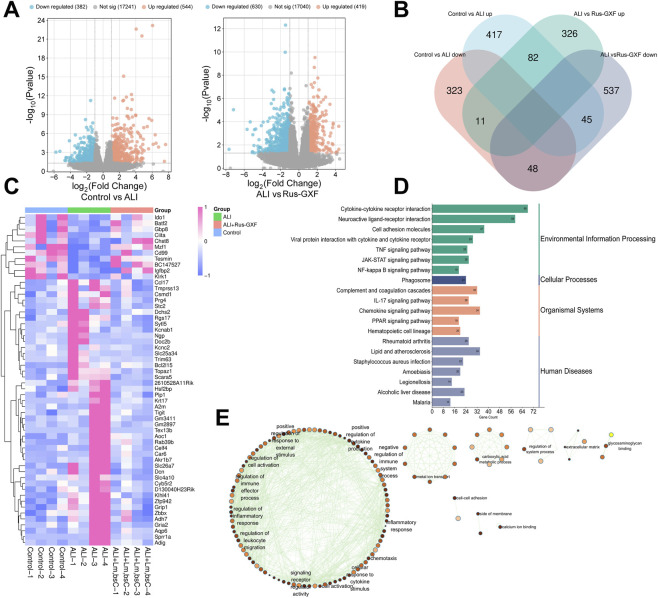
Transcriptomic analysis of lung tissue. **(A)** Gene volcano plots. **(B)** Wayne diagram of different comparison groups. **(C)** Differential gene clustering heat map. **(D)** KEGG analysis plot targeting differential genes. **(E)** Functional module diagram for GO enrichment analysis targeting differential genes.

We found a total of 1,049 differentially expressed genes (DEGs) in the Rus-GXF group compared to the ALI group, of which 419 genes were upregulated and 630 were downregulated, and 926 DEGs in the ALI group compared to the Control group, of which 544 were upregulated and 382 were downregulated (Figures 5A,B). We discovered that 11 genes in the ALI group exhibited substantial clustering separation from the ALI + Rus-GXF group, whereas 45 genes in the Control group exhibited significant clustering separation from the ALI group (Figure 5C). These pathways were primarily enriched in the modules for environmental information processing, cellular processes, the organismal system, and human diseases, according to KEGG enrichment analysis of all DEGs. We were shocked to see that the environmental information processing module had a substantially higher level of the JAK-STAT signaling pathway. In addition to affecting cellular functions like cell-to-cell adhesion, cell membrane flanking, calcium binding, and interfering with the transfer of metal ions, carboxylic acid metabolism, the extracellular matrix, glycosaminoglycan binding, and other molecular functions (Figure 5D). GO enrichment analysis showed that the altered genes may also be linked to immune system regulation, response to exogenous stimuli, inflammation regulation, and other biological processes (Figure 5E).

### 3.6 Rus-GXF can be strongly combined with JAK1

**FIGURE 6:**
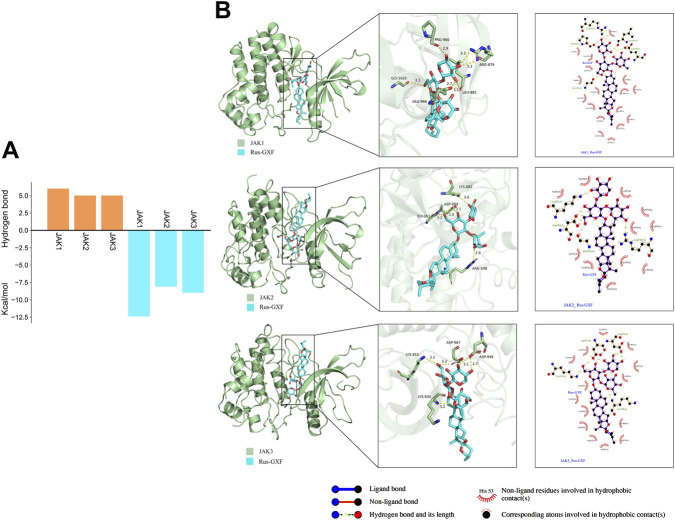
Molecular docking of Rus-GXF with JAKs-associated receptors. **(A)** Binding energy and hydrogen bond interactions between Rus-GXF and the receptors. **(B)** Three-dimensional representation of the binding conformation.

The JAKs receptors play a pivotal role in the regulation of inflammatory signaling pathways. Small-molecule binding to JAKs can modulate their downstream signal transduction. With a binding energy of −12.4 kcal/mol to JAK1, Rus-GXF had the highest binding capacity to JAK1, whereas the binding energies with JAK2 and JAK3 were −8.1 and −9 kcal/mol, respectively (Figure 6A). We discovered that Rus-GXF exhibited six hydrogen bond formations with JAK1, five with JAK2, and five with JAK3. Rus-GXF established two hydrogen bonds with ARG-879 and one with JAK1 at PRO-960, GLY-1020, GLU-996, and LEU-881. Five hydrogen bonds were established between Rus-GXF and JAK2 at LYS-882, ARG-938, GLY-993, and ASP-994. LYS-885, ASP-967, ASP-949, and LYS830 are the locations of the five hydrogen bonds that Rus-GXF and JAK3 formed respectively (Figure 6B).

**FIGURE 7:**
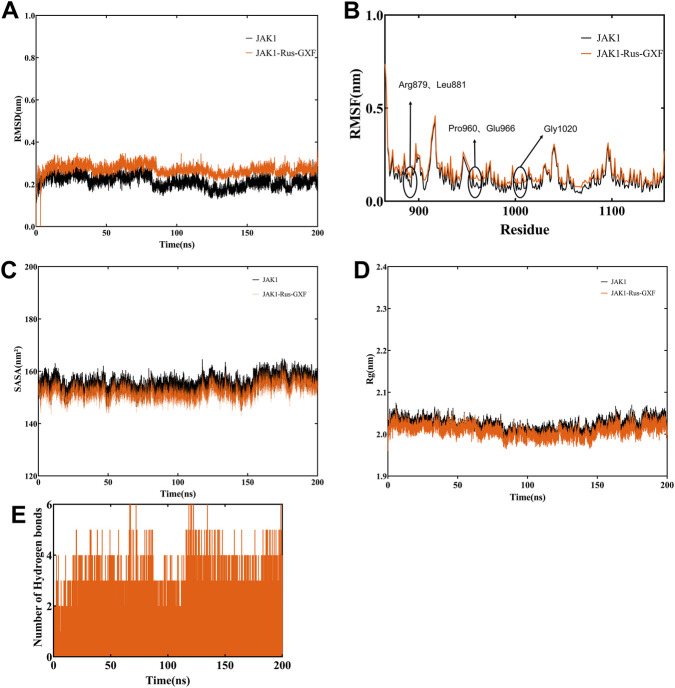
Molecular dynamics simulation analysis. **(A)** RMSD of Rus-GXF and JAK1 for 200 ns. **(B)** RMSF value of Rus-GXF and JAK1 for 200 ns. **(C)** Rg of Rus-GXF and JAK1 for 200 ns. **(D)** SASA value for all the systems of Rus-GXF and JAK1 for 200 ns. **(E)** Hydrogen bond of Rus-GXF and JAK1 for 200 ns.

A stable RMSD value indicates that the protein-ligand complex has reached conformational equilibrium during the simulation. Simulation analysis revealed that the RMSD of the complex remained stable with fluctuations less than 0.1 nm after 50 ns, showing no significant anomalous shifts (Figure 7A). The RMSF values for all residues were constrained within 1 nm, with no substantial fluctuations observed. Notably, the key residues within the JAK1 binding pocket (Arg879, Leu881, Pro960, Glu966, and Gly1020) all exhibited relatively low RMSF values (Figure 7B). The stable SASA implies maintained surface accessibility without major structural relaxation. In the SASA profile, the complex demonstrated minimal variation, with fluctuations within a 20 nm^2^ range and no pronounced sudden increases or decreases throughout the simulation (Figure 7C). The stability of the Rg reflects constant global protein compactness. Furthermore, the radius of gyration (Rg) displayed an overall decreasing trend while maintaining a stable and compact pattern during the entire MD simulation (Figure 7D). Consistent hydrogen bond formation, especially with key residues, confirms the sustained critical interactions between the ligand and target protein during simulation, directly evidencing stable binding. During the 100–200 ns time period, the complex maintained a high number of hydrogen bonds (Figure 7E).

### 3.7 Rus-GXF protects mice from ALI through modulation of the JAK1/STAT3 signaling pathway

**FIGURE 8:**
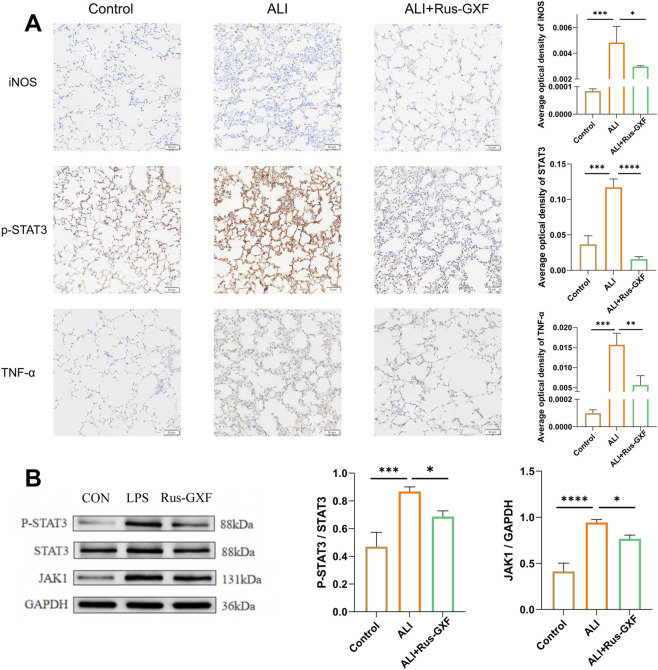
The verification of the JAK1/STAT3 signaling pathway. **(A)** The immunohistochemical staining assay was performed to investigate the expression levels of p-STAT3, TNF-α, and iNOS (*n* = 3). **(B)** Western blot analysis was conducted to assess both the expression level of JAK1 and the activation level of p-STAT3 (*n* = 3). Bars are expressed as mean ± SD. *P < 0.05; **P < 0.01; ***P < 0.001.

To further investigate the underlying signaling pathways, we performed immunohistochemistry (IHC) and Western blot analysis. Our results demonstrated that the expression of phosphorylated STAT3 (p-STAT3) was significantly elevated in the model group compared to the blank control group. However, treatment with Rus-GXF effectively suppressed p-STAT3 activation. Additionally, we observed upregulated expression of TNF-α and iNOS in the model group, indicating a robust inflammatory response (Figure 8A). Notably, Rus-GXF administration markedly reduced the levels of these pro-inflammatory mediators. Furthermore, both JAK1 protein expression and STAT3 phosphorylation levels were significantly altered, confirming the activation of the JAK1/STAT3 signaling pathway in the model group. These findings suggest that Rus-GXF exerts its anti-inflammatory effects, at least in part, by modulating the JAK1/STAT3 pathway (Figure 8B).

## 4 Discussion

In the Chinese Pharmacopoeia, Rus-GXF is the identification metabolite of *Liriope muscari* (Decaisne) L. H. Bailey, and its content change is closely related to the efficacy. However, its specific pharmacological activities, particularly against acute lung injury, remain largely unexplored. Given this gap, in this study, we preliminarily studied the anti-inflammatory activity of Rus-GXF *in vitro* and its protective effect on LPS-induced ALI mice.

ALI is usually caused by bacterial or viral infection (Dreyfuss and Ricard, 2005). The LPS component produced by pathogenic bacteria is an activator of many inflammation-related receptors, which can activate subsequent pathways, eventually leading to an increase in the secretion of inflammatory factors and causing serious tissue damage. Therefore, intratracheal instillation of LPS is also a common modeling method for the ALI mice model (Wang et al., 2008). Macrophages are the most common immune cells and play an important role in the progression of ALI (Laskin et al., 2019). Different forms of transformation of macrophages M1/M2 lead to different functions of pro-inflammatory or anti-inflammatory (Ni Gabhann et al., 2014). M1 macrophages can highly express iNOS and COX-2 genes and pro-inflammatory cytokines (such as IL-1β, IL-6, TNF-α), forming an inflammatory cascade, leading to alveolar-capillary barrier damage and pulmonary edema. Therefore, macrophages are the source cells of inflammatory cytokine storms (Yan et al., 2024). BEAS-2B cell-derived CXCL1 and CXCL2 act as chemoattractants for neutrophils (Sawant et al., 2023). Once recruited, neutrophils amplify the inflammatory response and release abundant matrix metalloproteinases and elastase, which degrade tight junction proteins (ZO-1, Occludin). ZO-1 is a crucial scaffolding protein that anchors transmembrane proteins to the cytoskeleton, while Occludin is a key transmembrane protein responsible for forming the paracellular seal. In ALI, pro-inflammatory cytokines disrupt barrier function by downregulating and dislocating these proteins, which ultimately accelerates lung pathology (Xiao et al., 2022; Woo et al., 2016; Kumar et al., 2018; Daly et al., 2016). Our study demonstrated that Rus-GXF not only modulated inflammatory responses in both macrophages and airway epithelial cells but also exerted cytoprotective effects.

The significance of this finding is underscored by the dual role of STAT3. Its activation not only controls pro-inflammatory transcription (Lee et al., 2024) but also contributes to barrier dysfunction by mediating the downregulation of key tight junction proteins (Yun et al., 2017; He et al., 2024; Li et al., 2021; Min et al., 2019), making JAK1 inhibition a pivotal intervention. The importance of JAK1 inhibition is well-acknowledged in the treatment of diverse inflammatory conditions, including rheumatoid arthritis, psoriasis, and pneumonia (Singh, 2019; Chen et al., 2024; Caso et al., 2023; He and Tian, 2021), which aligns with the mechanism identified in our study. Our screening revealed a set of core targets associated with ALI, which also exhibit interactions among themselves. Within this network, ESR1, functioning as a nuclear receptor, contributes to inflammation mainly via gene transcription regulation. Meanwhile, activated STAT3 is capable of binding to the ESR1 promoter and influencing its expression (Yamamoto et al., 2000; Niu et al., 2023). HIF1A serves as a key metabolic sensor that regulates immune cell function, pro-inflammatory cytokine release, and inflammatory processes. STAT3 can stabilize the HIF1A protein and promote its synthesis (Tang et al., 2023).

Currently available natural products that inhibit the JAK-STAT signaling pathway mainly include flavonoids, alkaloids, and terpenoids. Among these inhibitors, those targeting JAK1 and JAK2 significantly outnumber the inhibitors of JAK3 and TYK4 (Yang et al., 2025). Among these inhibitors, Cycloastragenol and Ouabain also belong to the triterpenoid and steroidal classes. Cycloastragenol demonstrates a potent inhibitory effect on JAK1 and, in combination with paclitaxel, exhibits significant cytotoxic efficacy against gastric adenocarcinoma cells (Hwang et al., 2019). Ouabain, a cardenolide characterized by a steroidal scaffold fused with a lactone ring, exhibits antiviral specificity through the selective downregulation of JAK1 via a Na^+^/K^+^-ATPase-independent proteolytic mechanism (Yang et al., 2020).

**FIGURE 9:**
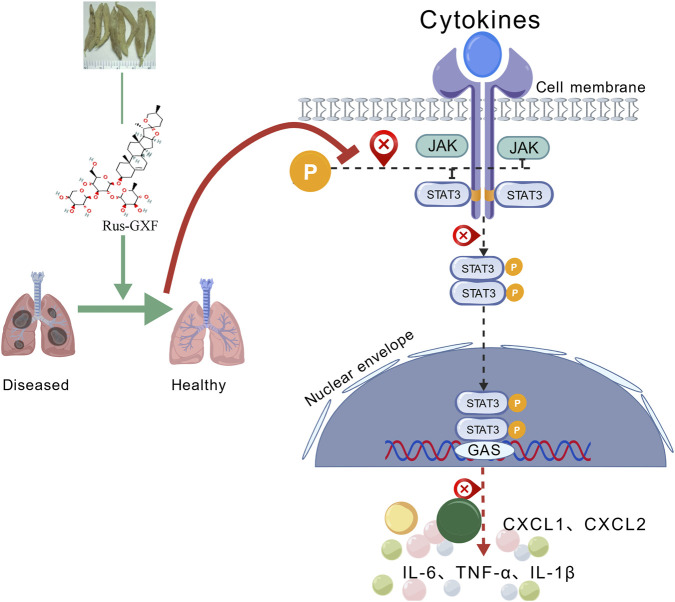
Mechanism diagram of intervention of Rus-GXF on ALI.

Molecular docking found that Rus-GXF has a high binding energy with JAK1, and its competitive inhibition of JAK1 can bloc k the activation of downstream pathways. The key binding residues of Rus-GXF within the JAK1 binding pocket include Arg879, Leu881, Pro960, Glu966, and Gly1020. Literature review and comparison with previous studies indicate that the binding pocket formed by these residues corresponds to the core binding region for its endogenous substrate, adenosine diphosphate (ADP) (Liau et al., 2018). Meanwhile, the low RMSF values of the key residues further validate the accuracy of the ADP-binding domain. In the normal catalytic cycle, the release of ADP is a prerequisite for the kinase to revert from a “closed” to an “open” state, a crucial step that prepares the enzyme for subsequent ATP binding. By occupying the ADP-binding pocket, a small molecule inhibitor obstructs this necessary conformational reset and thus prevents the initiation of a new phosphorylation cycle (Kavanagh et al., 2022). In this trapped, inactive conformation, the pocket is structurally more complementary to ADP than to ATP. Consequently, by binding to the ADP-binding site, Rus-GXF stabilizes JAK1 in an inactive state that is incompetent for ATP binding, achieving sustained inhibition (Vrontaki et al., 2017). Molecular docking results further revealed that, beyond forming specific interactions with the aforementioned key residues, Rus-GXF engages in extensive hydrophobic interactions with amino acid residues such as Phe, Asp, Gly, and Lys within the JAK1 binding pocket. As a major stabilizing force in biomolecular complexes, these hydrophobic interactions effectively reduce the solvation energy at the ligand-protein interface, thereby enhancing the binding stability of Rus-GXF within the JAK1 binding pocket. This binding mode aligns with the classical mechanism of kinase inhibitors, suggesting that Rus-GXF possesses targeted inhibitory activity against JAK1. The complex exhibited stable fluctuations in RMSD (<0.1 nm) after 50 ns of simulation, maintained a stable and compact conformation in Rg analysis, and showed low fluctuations in key residue RMSF. Additionally, both SASA and hydrogen bonds analyses indicated stable binding of the complex, further validating the conclusions derived from molecular docking. These findings provide a crucial theoretical foundation for subsequent studies, including *in vitro* enzyme inhibition assays and *in vivo* pharmacological evaluations (Figure 9).

While computational approaches like network pharmacology provide powerful starting hypotheses by predicting potential targets and pathways, their extrapolation to biological systems carries inherent assumptions. These include the static nature of protein structures in docking, the incompleteness of underlying databases, the risk of overlooking potential off-target effects, and the inability to model complex pharmacokinetics and dynamic protein-protein interactions *in vivo*. Importantly, our study was designed to address these limitations directly. The transcriptomic data validated the predicted pathway alterations at the whole-genome level, while the subsequent Western blot and immunohistochemical analyses confirmed the key protein-level changes in the relevant tissue context. Therefore, our integrated strategy mitigates the risk of over-relying on computational predictions alone.

For Rus-GXF *in vitro* experiments, cytotoxic effects were observed at different concentrations in the two cell lines due to their differential sensitivity. Furthermore, cytotoxicity under high-concentration, static *in vitro* conditions cannot directly predict *in vivo* toxicity. The *in vivo* environment involves dynamic pharmacokinetic processes, which typically result in significantly lower actual drug exposure at the target tissues compared to the administered dose, along with transient exposure duration. Therefore, its *in vivo* safety profile requires careful consideration.

Most current drugs are aimed at solely anti-inflammatory, like corticosteroids, overlooking the active restoration of the compromised alveolar-capillary barrier. Rus-GXF offers a potential clinical advantage due to its dual ability to reduce inflammation and enhance barrier integrity. However, further aspects must be rigorously evaluated to fully assess its translational potential. Regarding its toxicological evaluation, studies have systematically assessed the genotoxicity and subchronic toxicity of Rus-GXF. For instance, results from the Ames test and the mouse bone marrow micronucleus assay were both negative, indicating no mutagenic or clastogenic effects at the tested doses. In an acute toxicity study, a single oral dose as high as 5,000 mg/kg caused no mortality or significant abnormalities in mice, suggesting very low acute toxicity. In a subchronic toxicity experiment where rats were orally administered 10–360 mg/kg for 90 consecutive days, no treatment-related toxic changes were observed in hematological and biochemical parameters or histopathological examinations. However, common shortcomings of plant-derived saponins, such as poor oral absorption and low bioavailability, can severely limit their clinical route of administration and therapeutic efficacy, thereby directly impacting dosage regimen design. Addressing this challenge requires the exploration of novel drug delivery systems. Finally, comprehensive preclinical toxicological assessments (e.g., long-term toxicity, reproductive toxicity) and adherence to the specialized regulatory development pathway for botanical drugs are essential steps for its successful future translation (Ma et al., 2011).

## 5 Conclusion

Rus-GXF can regulate the inflammatory response and improve the function of BEAS-2B lung cells, thereby exerting a protective effect on mice with ALI. This mechanism involves the inhibition of the JAK1/STAT3 pathway.

## Data Availability

The transcriptomics data presented in the study are publicly available. This data can be found in the NCBI repository, accession number PRJNA1431556.
